# *mer*-(3,5-Dichloro-2-oxidobenzaldehyde thio­semicarbazonato-κ^3^ *S*,*N* ^1^,*O*)(methanol-κ*O*)(1,10-phenanthroline-κ^2^ *N*,*N*′)nickel(II)

**DOI:** 10.1107/S1600536809013208

**Published:** 2009-04-18

**Authors:** J. Y. Gao, Z. Liu, Y. Wang

**Affiliations:** a Key Laboratory of Non-ferrous Metal Materials and Processing Technology, Department of Material and Chemical Engineering, Guilin University of Technology, Ministry of Education, Guilin 541004, People’s Republic of China

## Abstract

In the title compound, [Ni(C_8_H_5_Cl_2_N_3_OS)(C_12_H_8_N_2_)(CH_3_OH)], the Ni^II^ atom is octa­hedrally coordinated by one N, one O and one S atom from a 3,5-dichloro-2-oxidobenzaldehyde thio­semicarbazonate ligand, another O atom from methanol and another two N atoms from 1,10-phenanthroline. The crystal structure is constructed by N—H⋯Cl, N—H⋯N, C—H⋯S and O—H⋯S hydrogen bonds.

## Related literature

For nickel complexes with salicylic aldehyde thio­semi­carbazone ligands, see: Dapporto *et al.* (1984 ▶); Schulte *et al.* (1991 ▶); García-Reynaldos *et al.* (2007 ▶); Kolotilov *et al.* (2007 ▶); Qiu & Wu (2004 ▶). For related Cu(II) compounds with a distorted octahedral coordination as a result of the Jahn–Teller effect, see: García-Orozco *et al.* (2002 ▶). For bond-length data, see: Orpen *et al.* (1989 ▶). For related structures, see: Seena & Kurup (2007 ▶); Wang *et al.* (2008 ▶); Zhang *et al.* (2007 ▶).
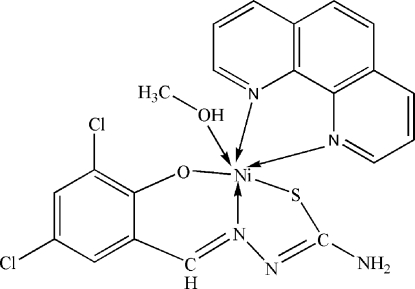


## Experimental

### 

#### Crystal data

[Ni(C_8_H_5_Cl_2_N_3_OS)(C_12_H_8_N_2_)(CH_4_O)]
*M* *_r_* = 533.07
Monoclinic, 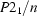

*a* = 12.058 (1) Å
*b* = 12.946 (1) Å
*c* = 14.973 (2) Å
β = 105.918 (1)°
*V* = 2247.6 (4) Å^3^
*Z* = 4
Mo *K*α radiation
μ = 1.22 mm^−1^
*T* = 298 K
0.30 × 0.28 × 0.13 mm

#### Data collection

Bruker SMART CCD area-detector diffractometer
Absorption correction: multi-scan (*SADABS*; Sheldrick, 1996 ▶) *T*_min_ = 0.710, *T*_max_ = 0.857
10910 measured reflections
3951 independent reflections
2708 reflections with *I* > 2σ(*I*)
*R*_int_ = 0.040

#### Refinement

*R*[*F* ^2^ > 2σ(*F* ^2^)] = 0.040
*wR*(*F* ^2^) = 0.119
*S* = 1.06
3951 reflections
289 parameters
H-atom parameters constrained
Δρ_max_ = 0.64 e Å^−3^
Δρ_min_ = −0.37 e Å^−3^

### 

Data collection: *SMART* (Bruker, 2001 ▶); cell refinement: *SAINT* (Bruker, 2001 ▶); data reduction: *SAINT*; program(s) used to solve structure: *SHELXS97* (Sheldrick, 2008 ▶); program(s) used to refine structure: *SHELXL97* (Sheldrick, 2008 ▶); molecular graphics: *SHELXTL* (Sheldrick, 2008 ▶); software used to prepare material for publication: *SHELXTL*.

## Supplementary Material

Crystal structure: contains datablocks global, I. DOI: 10.1107/S1600536809013208/im2109sup1.cif

Structure factors: contains datablocks I. DOI: 10.1107/S1600536809013208/im2109Isup2.hkl

Additional supplementary materials:  crystallographic information; 3D view; checkCIF report

## Figures and Tables

**Table 1:** Hydrogen-bond geometry (Å, °)

*D*—H⋯*A*	*D*—H	H⋯*A*	*D*⋯*A*	*D*—H⋯*A*
O2—H2⋯S1^i^	0.82	2.49	3.310 (3)	174
N3—H3*A*⋯N2^ii^	0.86	2.24	3.087 (4)	170
N3—H3*B*⋯Cl1^iii^	0.86	2.86	3.573 (2)	142
C11—H11⋯S1^iv^	0.93	2.81	3.593 (5)	142

Symmetry codes: (i) 
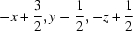
; (ii) 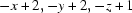; (iii) 
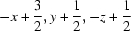
; (iv) 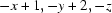.

**Fig. 1.**
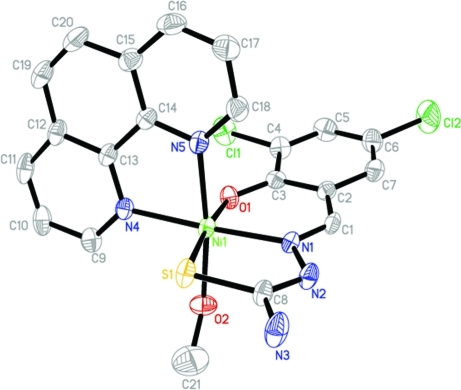
The molecular structure of (I), showing 30% probability displacement ellipsoids. Carbon-bound H atoms have been omitted.

**Fig. 2.**
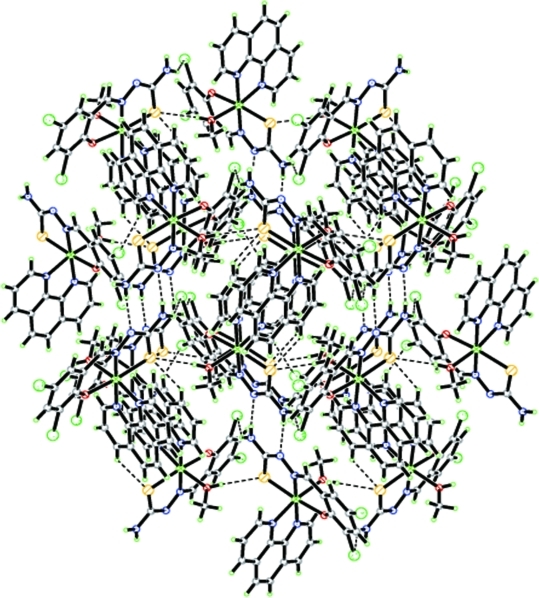
Three-dimensional network of (I), broken lines show N–H···Cl, N–H···N, C–H···S and O–H···S hydrogen bonds.

## supplementary crystallographic information

### Comment

As a special kind of Schiff bases, thiosemicarbazones and their metal complexes have become the subjects of intensive study because of their wide ranging biological activities, analytical applications and interesting chemical and structural properties. By now there are not many nickel complexes with salicylic aldehyde thiosemicarbazone ligands [Dapporto *et al.* (1984); Schulte *et al.* (1991); García-Reynaldos *et al.* (2007); Kolotilov *et al.* (2007); Qiu *et al.* (2004)]. The additional use of 1,10-phenanthroline as the third ligand depicts another structural type.

In (I), the Ni^II^ atom is coordinated by one N, one O and one S atom from the tridentate dianionic 3,5-dichlorosalicylaldehyde thiosemicarbazonato ligand, one O atom from methanol and two N atoms from phen. The six atoms form a distorted octahedral coordination sphere around the metal because of Jahn-Teller effect (García-Orozco *et al.*, 2002). The Ni—S bond length is 2.358 (1) Å, which is very close to 2.295Å (Orpen *et al.* 1989). The three-dimensional network of (I) is established by N–H···Cl, N–H···N, C–H···S and O–H···S hydrogen bonds (Fig.2).

### Experimental

A solution of 3,5-dichlorosalicylaldehyde (10 mmol) in EtOH (30 ml) was added dropwise to an aqueous solution (25 ml) of thiosemicarbazide (10 mmol) and 1.5 ml acetic anhydride with stirring at *ca* 70° C for 4.5 h. The light brown precipitate was removed by filtration and recrystallized from 1:1 (*v*/*v*) MeOH/EtOH. Then a mixture of the ligand (1 mmol) and nickel nitrate (1 mmol) in MeOH (35 ml) was stirred at *ca* 65° C for 2 h. After 1,10-phenanthroline (1 mmol) was added to the mixture heating was continued for another 2 h. The Ni complex was dissolved in DMF and the resulting red solution was filtrated. After 4 days, red block crystals were obtained by slow evaporation of the solvent from the filtrate.

### Refinement

All the H atoms were placed in geometrically idealized positions and constrained to ride on their parent atoms, with C-H distances of 0.93-0.96Å, N-H distances of 0.86Å and O-H distances of 0.82Å,respectively, and Uiso(H) = 1.2-1.5*U*_eq_(C), Uiso(H) = 1.2*U*_eq_(N) and Uiso(H) = 1.5*U*_eq_(O).

### Crystal data

**Table:**

[Ni(C_8_H_5_Cl_2_N_3_OS)(C_12_H_8_N_2_)(CH_4_O)]	*F*(000) = 1088
*M**_r_* = 533.07	*D*_x_ = 1.575 Mg m^−^^3^
Monoclinic, *P*2_1_/*n*	Mo *K*α radiation, λ = 0.71073 Å
*a* = 12.058 (1) Å	Cell parameters from 3309 reflections
*b* = 12.946 (1) Å	θ = 3.6–25.3°
*c* = 14.973 (2) Å	µ = 1.22 mm^−^^1^
β = 105.918 (1)°	*T* = 298 K
*V* = 2247.6 (4) Å^3^	Block, red
*Z* = 4	0.30 × 0.28 × 0.13 mm

### Data collection

**Table:**

Bruker SMART CCD area-detector diffractometer	3951 independent reflections
Radiation source: fine-focus sealed tube	2708 reflections with *I* > 2σ(*I*)
graphite	*R*_int_ = 0.040
φ and ω scans	θ_max_ = 25.0°, θ_min_ = 2.1°
Absorption correction: multi-scan (*SADABS*; Sheldrick, 1996)	*h* = −13→14
*T*_min_ = 0.710, *T*_max_ = 0.857	*k* = −15→14
10910 measured reflections	*l* = −17→17

### Refinement

**Table:**

Refinement on *F*^2^	Primary atom site location: structure-invariant direct methods
Least-squares matrix: full	Secondary atom site location: difference Fourier map
*R*[*F*^2^ > 2σ(*F*^2^)] = 0.040	Hydrogen site location: inferred from neighbouring sites
*wR*(*F*^2^) = 0.119	H-atom parameters constrained
*S* = 1.06	*w* = 1/[σ^2^(*F*_o_^2^) + (0.0487*P*)^2^ + 1.8417*P*] where *P* = (*F*_o_^2^ + 2*F*_c_^2^)/3
3951 reflections	(Δ/σ)_max_ = 0.001
289 parameters	Δρ_max_ = 0.64 e Å^−^^3^
0 restraints	Δρ_min_ = −0.37 e Å^−^^3^

### Special details

**Table:**

Geometry. All e.s.d.'s (except the e.s.d. in the dihedral angle between two l.s. planes) are estimated using the full covariance matrix. The cell e.s.d.'s are taken into account individually in the estimation of e.s.d.'s in distances, angles and torsion angles; correlations between e.s.d.'s in cell parameters are only used when they are defined by crystal symmetry. An approximate (isotropic) treatment of cell e.s.d.'s is used for estimating e.s.d.'s involving l.s. planes.
Refinement. Refinement of *F*^2^ against ALL reflections. The weighted *R*-factor *wR* and goodness of fit *S* are based on *F*^2^, conventional *R*-factors *R* are based on *F*, with *F* set to zero for negative *F*^2^. The threshold expression of *F*^2^ > σ(*F*^2^) is used only for calculating *R*-factors(gt) *etc*. and is not relevant to the choice of reflections for refinement. *R*-factors based on *F*^2^ are statistically about twice as large as those based on *F*, and *R*- factors based on ALL data will be even larger.

### Fractional atomic coordinates and isotropic or equivalent isotropic displacement parameters (Å^2^)

**Table:**

	*x*	*y*	*z*	*U*_iso_*/*U*_eq_	
Ni1	0.79152 (4)	0.87614 (4)	0.19294 (3)	0.03473 (17)	
S1	0.73435 (9)	1.01246 (8)	0.27489 (7)	0.0455 (3)	
Cl1	0.88829 (11)	0.63336 (11)	−0.01564 (9)	0.0725 (4)	
Cl2	1.32931 (13)	0.62176 (14)	0.18947 (12)	0.0989 (6)	
N1	0.9387 (3)	0.8831 (2)	0.2981 (2)	0.0352 (7)	
N2	0.9476 (3)	0.9456 (3)	0.3753 (2)	0.0431 (8)	
N3	0.8598 (3)	1.0689 (3)	0.4404 (2)	0.0646 (12)	
H3A	0.9190	1.0694	0.4881	0.077*	
H3B	0.8024	1.1090	0.4385	0.077*	
N4	0.6453 (3)	0.8694 (2)	0.0803 (2)	0.0362 (7)	
N5	0.8398 (3)	0.9774 (2)	0.1016 (2)	0.0400 (8)	
O1	0.8512 (2)	0.7563 (2)	0.13516 (18)	0.0420 (7)	
O2	0.7231 (3)	0.7590 (2)	0.2641 (2)	0.0538 (8)	
H2	0.7287	0.6980	0.2512	0.081*	
C1	1.0316 (3)	0.8325 (3)	0.3000 (3)	0.0394 (10)	
H1	1.0940	0.8421	0.3519	0.047*	
C2	1.0484 (3)	0.7622 (3)	0.2296 (3)	0.0382 (9)	
C3	0.9579 (3)	0.7308 (3)	0.1505 (3)	0.0378 (9)	
C4	0.9926 (4)	0.6668 (3)	0.0864 (3)	0.0483 (11)	
C5	1.1037 (4)	0.6326 (4)	0.0978 (3)	0.0584 (12)	
H5	1.1217	0.5898	0.0539	0.070*	
C6	1.1881 (4)	0.6634 (4)	0.1761 (3)	0.0579 (12)	
C7	1.1614 (4)	0.7264 (3)	0.2404 (3)	0.0503 (11)	
H7	1.2194	0.7461	0.2926	0.060*	
C8	0.8571 (3)	1.0056 (3)	0.3689 (3)	0.0418 (10)	
C9	0.5507 (4)	0.8126 (3)	0.0704 (3)	0.0480 (11)	
H9	0.5425	0.7747	0.1209	0.058*	
C10	0.4635 (4)	0.8078 (4)	−0.0128 (3)	0.0587 (13)	
H10	0.3990	0.7665	−0.0175	0.070*	
C11	0.4735 (4)	0.8640 (3)	−0.0866 (3)	0.0560 (13)	
H11	0.4154	0.8615	−0.1423	0.067*	
C12	0.5710 (4)	0.9260 (3)	−0.0794 (3)	0.0460 (11)	
C13	0.6555 (3)	0.9246 (3)	0.0063 (3)	0.0371 (9)	
C14	0.7589 (3)	0.9836 (3)	0.0181 (3)	0.0385 (9)	
C15	0.7722 (4)	1.0463 (3)	−0.0550 (3)	0.0501 (11)	
C16	0.8737 (5)	1.1054 (4)	−0.0371 (3)	0.0611 (13)	
H16	0.8860	1.1489	−0.0829	0.073*	
C17	0.9543 (4)	1.0995 (4)	0.0467 (4)	0.0631 (14)	
H17	1.0213	1.1387	0.0588	0.076*	
C18	0.9340 (4)	1.0331 (4)	0.1140 (3)	0.0532 (12)	
H18	0.9900	1.0281	0.1706	0.064*	
C19	0.5891 (4)	0.9883 (4)	−0.1528 (3)	0.0591 (13)	
H19	0.5339	0.9884	−0.2101	0.071*	
C20	0.6836 (5)	1.0465 (4)	−0.1410 (3)	0.0614 (13)	
H20	0.6918	1.0879	−0.1896	0.074*	
C21	0.6611 (6)	0.7670 (5)	0.3301 (5)	0.108 (2)	
H21A	0.7122	0.7870	0.3888	0.162*	
H21B	0.6267	0.7015	0.3365	0.162*	
H21C	0.6017	0.8181	0.3105	0.162*	

### Atomic displacement parameters (Å^2^)

**Table:**

	*U*^11^	*U*^22^	*U*^33^	*U*^12^	*U*^13^	*U*^23^
Ni1	0.0340 (3)	0.0319 (3)	0.0326 (3)	0.0002 (2)	−0.0005 (2)	−0.0013 (2)
S1	0.0426 (6)	0.0409 (6)	0.0424 (6)	0.0096 (5)	−0.0065 (5)	−0.0087 (5)
Cl1	0.0662 (8)	0.0739 (9)	0.0733 (9)	−0.0054 (7)	0.0119 (6)	−0.0356 (7)
Cl2	0.0624 (9)	0.1247 (14)	0.1059 (12)	0.0483 (9)	0.0165 (8)	−0.0035 (10)
N1	0.0361 (18)	0.0347 (18)	0.0302 (16)	0.0029 (15)	0.0015 (13)	0.0019 (14)
N2	0.043 (2)	0.044 (2)	0.0340 (18)	0.0084 (16)	−0.0028 (15)	−0.0093 (15)
N3	0.060 (2)	0.074 (3)	0.047 (2)	0.022 (2)	−0.0076 (18)	−0.026 (2)
N4	0.0354 (18)	0.0324 (18)	0.0366 (18)	0.0010 (15)	0.0028 (14)	−0.0042 (15)
N5	0.0381 (18)	0.0372 (19)	0.0400 (19)	−0.0003 (15)	0.0025 (15)	0.0009 (15)
O1	0.0351 (16)	0.0375 (16)	0.0496 (16)	−0.0010 (12)	0.0051 (12)	−0.0068 (13)
O2	0.071 (2)	0.0428 (18)	0.0553 (19)	−0.0042 (16)	0.0301 (16)	0.0033 (15)
C1	0.034 (2)	0.045 (2)	0.031 (2)	0.0009 (19)	−0.0040 (17)	0.0041 (18)
C2	0.041 (2)	0.033 (2)	0.039 (2)	0.0036 (18)	0.0088 (18)	0.0083 (17)
C3	0.044 (2)	0.027 (2)	0.044 (2)	−0.0016 (18)	0.0137 (19)	0.0038 (17)
C4	0.053 (3)	0.035 (2)	0.056 (3)	−0.001 (2)	0.012 (2)	−0.007 (2)
C5	0.064 (3)	0.047 (3)	0.070 (3)	0.011 (2)	0.028 (3)	−0.007 (2)
C6	0.047 (3)	0.058 (3)	0.069 (3)	0.018 (2)	0.016 (2)	0.009 (3)
C7	0.046 (3)	0.051 (3)	0.050 (3)	0.011 (2)	0.006 (2)	0.010 (2)
C8	0.040 (2)	0.041 (2)	0.037 (2)	0.0004 (19)	−0.0015 (18)	−0.0050 (18)
C9	0.043 (3)	0.042 (3)	0.056 (3)	−0.003 (2)	0.008 (2)	−0.006 (2)
C10	0.038 (3)	0.054 (3)	0.070 (3)	−0.003 (2)	−0.009 (2)	−0.013 (3)
C11	0.047 (3)	0.050 (3)	0.053 (3)	0.011 (2)	−0.017 (2)	−0.016 (2)
C12	0.049 (3)	0.045 (3)	0.035 (2)	0.010 (2)	−0.0034 (19)	−0.0093 (19)
C13	0.043 (2)	0.030 (2)	0.034 (2)	0.0076 (18)	0.0038 (18)	−0.0036 (17)
C14	0.046 (2)	0.033 (2)	0.035 (2)	0.0056 (18)	0.0080 (18)	−0.0007 (17)
C15	0.058 (3)	0.044 (3)	0.049 (3)	0.008 (2)	0.016 (2)	0.007 (2)
C16	0.076 (4)	0.052 (3)	0.063 (3)	0.004 (3)	0.031 (3)	0.018 (2)
C17	0.060 (3)	0.051 (3)	0.077 (4)	−0.013 (2)	0.017 (3)	0.009 (3)
C18	0.045 (3)	0.054 (3)	0.054 (3)	−0.009 (2)	0.003 (2)	0.002 (2)
C19	0.070 (3)	0.061 (3)	0.036 (2)	0.015 (3)	−0.004 (2)	−0.002 (2)
C20	0.080 (4)	0.064 (3)	0.037 (3)	0.019 (3)	0.012 (2)	0.012 (2)
C21	0.132 (6)	0.090 (5)	0.108 (5)	−0.016 (4)	0.043 (5)	0.021 (4)

### Geometric parameters (Å, °)

**Table:**

Ni1—O1	2.004 (3)	C5—C6	1.383 (6)
Ni1—N1	2.026 (3)	C5—H5	0.9300
Ni1—N4	2.081 (3)	C6—C7	1.366 (6)
Ni1—N5	2.089 (3)	C7—H7	0.9300
Ni1—O2	2.145 (3)	C9—C10	1.393 (6)
Ni1—S1	2.3578 (11)	C9—H9	0.9300
S1—C8	1.743 (4)	C10—C11	1.357 (7)
Cl1—C4	1.746 (4)	C10—H10	0.9300
Cl2—C6	1.745 (5)	C11—C12	1.403 (6)
N1—C1	1.290 (5)	C11—H11	0.9300
N1—N2	1.391 (4)	C12—C13	1.403 (5)
N2—C8	1.321 (5)	C12—C19	1.427 (6)
N3—C8	1.342 (5)	C13—C14	1.430 (5)
N3—H3A	0.8600	C14—C15	1.406 (6)
N3—H3B	0.8600	C15—C16	1.405 (6)
N4—C9	1.331 (5)	C15—C20	1.430 (6)
N4—C13	1.353 (5)	C16—C17	1.362 (6)
N5—C18	1.314 (5)	C16—H16	0.9300
N5—C14	1.362 (5)	C17—C18	1.396 (6)
O1—C3	1.286 (4)	C17—H17	0.9300
O2—C21	1.398 (7)	C18—H18	0.9300
O2—H2	0.8200	C19—C20	1.338 (7)
C1—C2	1.448 (5)	C19—H19	0.9300
C1—H1	0.9300	C20—H20	0.9300
C2—C7	1.407 (5)	C21—H21A	0.9600
C2—C3	1.433 (5)	C21—H21B	0.9600
C3—C4	1.415 (6)	C21—H21C	0.9600
C4—C5	1.376 (6)		
			
O1—Ni1—N1	91.59 (11)	C5—C6—Cl2	118.4 (4)
O1—Ni1—N4	86.69 (11)	C6—C7—C2	121.6 (4)
N1—Ni1—N4	177.11 (12)	C6—C7—H7	119.2
O1—Ni1—N5	90.32 (12)	C2—C7—H7	119.2
N1—Ni1—N5	97.97 (12)	N2—C8—N3	117.6 (3)
N4—Ni1—N5	79.74 (12)	N2—C8—S1	126.2 (3)
O1—Ni1—O2	84.23 (11)	N3—C8—S1	116.3 (3)
N1—Ni1—O2	91.15 (12)	N4—C9—C10	122.5 (4)
N4—Ni1—O2	90.98 (12)	N4—C9—H9	118.7
N5—Ni1—O2	169.51 (12)	C10—C9—H9	118.7
O1—Ni1—S1	174.34 (8)	C11—C10—C9	119.4 (4)
N1—Ni1—S1	83.20 (9)	C11—C10—H10	120.3
N4—Ni1—S1	98.62 (9)	C9—C10—H10	120.3
N5—Ni1—S1	92.58 (10)	C10—C11—C12	120.2 (4)
O2—Ni1—S1	93.65 (9)	C10—C11—H11	119.9
C8—S1—Ni1	94.43 (14)	C12—C11—H11	119.9
C1—N1—N2	114.1 (3)	C13—C12—C11	116.5 (4)
C1—N1—Ni1	124.4 (3)	C13—C12—C19	119.0 (4)
N2—N1—Ni1	121.5 (2)	C11—C12—C19	124.4 (4)
C8—N2—N1	114.1 (3)	N4—C13—C12	123.3 (4)
C8—N3—H3A	120.0	N4—C13—C14	116.9 (3)
C8—N3—H3B	120.0	C12—C13—C14	119.7 (4)
H3A—N3—H3B	120.0	N5—C14—C15	122.9 (4)
C9—N4—C13	118.0 (3)	N5—C14—C13	117.5 (3)
C9—N4—Ni1	128.5 (3)	C15—C14—C13	119.6 (4)
C13—N4—Ni1	113.3 (2)	C16—C15—C14	116.4 (4)
C18—N5—C14	118.1 (4)	C16—C15—C20	124.5 (4)
C18—N5—Ni1	129.5 (3)	C14—C15—C20	119.0 (4)
C14—N5—Ni1	112.3 (3)	C17—C16—C15	120.6 (4)
C3—O1—Ni1	125.6 (2)	C17—C16—H16	119.7
C21—O2—Ni1	130.7 (3)	C15—C16—H16	119.7
C21—O2—H2	109.5	C16—C17—C18	118.5 (4)
Ni1—O2—H2	119.7	C16—C17—H17	120.7
N1—C1—C2	126.6 (3)	C18—C17—H17	120.7
N1—C1—H1	116.7	N5—C18—C17	123.4 (4)
C2—C1—H1	116.7	N5—C18—H18	118.3
C7—C2—C3	119.7 (4)	C17—C18—H18	118.3
C7—C2—C1	116.6 (4)	C20—C19—C12	121.5 (4)
C3—C2—C1	123.6 (4)	C20—C19—H19	119.3
O1—C3—C4	119.8 (4)	C12—C19—H19	119.3
O1—C3—C2	124.9 (4)	C19—C20—C15	121.1 (4)
C4—C3—C2	115.3 (4)	C19—C20—H20	119.5
C5—C4—C3	124.2 (4)	C15—C20—H20	119.5
C5—C4—Cl1	118.2 (3)	O2—C21—H21A	109.5
C3—C4—Cl1	117.5 (3)	O2—C21—H21B	109.5
C4—C5—C6	118.5 (4)	H21A—C21—H21B	109.5
C4—C5—H5	120.7	O2—C21—H21C	109.5
C6—C5—H5	120.7	H21A—C21—H21C	109.5
C7—C6—C5	120.6 (4)	H21B—C21—H21C	109.5
C7—C6—Cl2	121.0 (4)		
			
O1—Ni1—S1—C8	17.3 (9)	C7—C2—C3—C4	−2.1 (5)
N1—Ni1—S1—C8	−5.61 (16)	C1—C2—C3—C4	176.2 (4)
N4—Ni1—S1—C8	176.66 (17)	O1—C3—C4—C5	−177.9 (4)
N5—Ni1—S1—C8	−103.33 (16)	C2—C3—C4—C5	2.0 (6)
O2—Ni1—S1—C8	85.12 (16)	O1—C3—C4—Cl1	4.5 (5)
O1—Ni1—N1—C1	10.5 (3)	C2—C3—C4—Cl1	−175.6 (3)
N4—Ni1—N1—C1	−43 (3)	C3—C4—C5—C6	−1.0 (7)
N5—Ni1—N1—C1	−80.1 (3)	Cl1—C4—C5—C6	176.6 (4)
O2—Ni1—N1—C1	94.7 (3)	C4—C5—C6—C7	−0.1 (7)
S1—Ni1—N1—C1	−171.7 (3)	C4—C5—C6—Cl2	−178.9 (4)
O1—Ni1—N1—N2	−170.2 (3)	C5—C6—C7—C2	−0.1 (7)
N4—Ni1—N1—N2	137 (2)	Cl2—C6—C7—C2	178.7 (3)
N5—Ni1—N1—N2	99.2 (3)	C3—C2—C7—C6	1.3 (6)
O2—Ni1—N1—N2	−86.0 (3)	C1—C2—C7—C6	−177.1 (4)
S1—Ni1—N1—N2	7.6 (3)	N1—N2—C8—N3	−179.3 (4)
C1—N1—N2—C8	173.2 (3)	N1—N2—C8—S1	−0.6 (5)
Ni1—N1—N2—C8	−6.2 (4)	Ni1—S1—C8—N2	5.3 (4)
O1—Ni1—N4—C9	87.0 (3)	Ni1—S1—C8—N3	−175.9 (3)
N1—Ni1—N4—C9	140 (2)	C13—N4—C9—C10	0.4 (6)
N5—Ni1—N4—C9	177.9 (4)	Ni1—N4—C9—C10	−173.5 (3)
O2—Ni1—N4—C9	2.8 (3)	N4—C9—C10—C11	−1.0 (7)
S1—Ni1—N4—C9	−91.0 (3)	C9—C10—C11—C12	0.3 (7)
O1—Ni1—N4—C13	−87.1 (3)	C10—C11—C12—C13	0.9 (6)
N1—Ni1—N4—C13	−34 (3)	C10—C11—C12—C19	−179.6 (4)
N5—Ni1—N4—C13	3.8 (3)	C9—N4—C13—C12	0.9 (6)
O2—Ni1—N4—C13	−171.3 (3)	Ni1—N4—C13—C12	175.7 (3)
S1—Ni1—N4—C13	94.8 (2)	C9—N4—C13—C14	−179.0 (3)
O1—Ni1—N5—C18	−96.3 (4)	Ni1—N4—C13—C14	−4.1 (4)
N1—Ni1—N5—C18	−4.6 (4)	C11—C12—C13—N4	−1.6 (6)
N4—Ni1—N5—C18	177.2 (4)	C19—C12—C13—N4	178.9 (4)
O2—Ni1—N5—C18	−154.8 (6)	C11—C12—C13—C14	178.3 (4)
S1—Ni1—N5—C18	78.9 (4)	C19—C12—C13—C14	−1.2 (6)
O1—Ni1—N5—C14	83.7 (3)	C18—N5—C14—C15	0.4 (6)
N1—Ni1—N5—C14	175.4 (3)	Ni1—N5—C14—C15	−179.6 (3)
N4—Ni1—N5—C14	−2.8 (3)	C18—N5—C14—C13	−178.5 (4)
O2—Ni1—N5—C14	25.2 (8)	Ni1—N5—C14—C13	1.5 (4)
S1—Ni1—N5—C14	−101.1 (3)	N4—C13—C14—N5	1.8 (5)
N1—Ni1—O1—C3	−20.1 (3)	C12—C13—C14—N5	−178.1 (4)
N4—Ni1—O1—C3	157.6 (3)	N4—C13—C14—C15	−177.2 (4)
N5—Ni1—O1—C3	77.9 (3)	C12—C13—C14—C15	3.0 (6)
O2—Ni1—O1—C3	−111.1 (3)	N5—C14—C15—C16	−1.5 (6)
S1—Ni1—O1—C3	−42.8 (10)	C13—C14—C15—C16	177.4 (4)
O1—Ni1—O2—C21	178.3 (5)	N5—C14—C15—C20	178.8 (4)
N1—Ni1—O2—C21	86.9 (5)	C13—C14—C15—C20	−2.3 (6)
N4—Ni1—O2—C21	−95.1 (5)	C14—C15—C16—C17	1.0 (7)
N5—Ni1—O2—C21	−122.7 (7)	C20—C15—C16—C17	−179.3 (5)
S1—Ni1—O2—C21	3.6 (5)	C15—C16—C17—C18	0.3 (7)
N2—N1—C1—C2	−179.7 (4)	C14—N5—C18—C17	1.1 (7)
Ni1—N1—C1—C2	−0.3 (6)	Ni1—N5—C18—C17	−178.9 (3)
N1—C1—C2—C7	171.7 (4)	C16—C17—C18—N5	−1.5 (8)
N1—C1—C2—C3	−6.6 (6)	C13—C12—C19—C20	−1.2 (7)
Ni1—O1—C3—C4	−160.4 (3)	C11—C12—C19—C20	179.3 (4)
Ni1—O1—C3—C2	19.7 (5)	C12—C19—C20—C15	1.9 (7)
C7—C2—C3—O1	177.8 (4)	C16—C15—C20—C19	−179.8 (5)
C1—C2—C3—O1	−3.9 (6)	C14—C15—C20—C19	−0.1 (7)

### Hydrogen-bond geometry (Å, °)

**Table:**

*D*—H···*A*	*D*—H	H···*A*	*D*···*A*	*D*—H···*A*
O2—H2···S1^i^	0.82	2.49	3.310 (3)	174
N3—H3A···N2^ii^	0.86	2.24	3.087 (4)	170
N3—H3B···Cl1^iii^	0.86	2.86	3.573 (2)	142
C11—H11···S1^iv^	0.93	2.81	3.593 (5)	142

Symmetry codes: (i) −*x*+3/2, *y*−1/2, −*z*+1/2; (ii) −*x*+2, −*y*+2, −*z*+1; (iii) −*x*+3/2, *y*+1/2, −*z*+1/2; (iv) −*x*+1, −*y*+2, −*z*.
